# Disinfection of the Water Borne Pathogens *Escherichia coli* and *Staphylococcus aureus* by Solar Photocatalysis Using Sonochemically Synthesized Reusable Ag@ZnO Core-Shell Nanoparticles

**DOI:** 10.3390/ijerph14070747

**Published:** 2017-07-10

**Authors:** Sourav Das, Neha Ranjana, Ananyo Jyoti Misra, Mrutyunjay Suar, Amrita Mishra, Ashok J. Tamhankar, Cecilia Stålsby Lundborg, Suraj K. Tripathy

**Affiliations:** 1 School of Biotechnology, Kalinga Institute of Industrial Technology (KIIT University), Bhubaneswar 751024, India; sddassourav52@gmail.com (S.D.); ranjana.nh@gmail.com (N.R.); ananyomisra@gmail.com (A.J.M.); msbiotek@yahoo.com (M.S.); amritamishrabio@gmail.com (A.M.); 2 Department of Public Health Sciences, Karolinska Institutet, SE 17177 Stockholm, Sweden; cecilia.stalsby.lundborg@ki.se; 3 School of Chemical Technology, KIIT University, Bhubaneswar 751024, India

**Keywords:** core-shell, disinfection, *Escherichia coli*, nanoparticles, pathogens, silver, solar-photocatalysis, *Staphylococcus aureus*, water, zinc oxide

## Abstract

Water borne pathogens present a threat to human health and their disinfection from water poses a challenge, prompting the search for newer methods and newer materials. Disinfection of the Gram-negative bacterium *Escherichia coli* and the Gram-positive coccal bacterium *Staphylococcus aureus* in an aqueous matrix was achieved within 60 and 90 min, respectively, at 35 °C using solar-photocatalysis mediated by sonochemically synthesized Ag@ZnO core-shell nanoparticles. The efficiency of the process increased with the increase in temperature and at 55 °C the disinfection for the two bacteria could be achieved in 45 and 60 min, respectively. A new ultrasound-assisted chemical precipitation technique was used for the synthesis of Ag@ZnO core-shell nanoparticles. The characteristics of the synthesized material were established using physical techniques. The material remained stable even at 400 °C. Disinfection efficiency of the Ag@ZnO core-shell nanoparticles was confirmed in the case of real world samples of pond, river, municipal tap water and was found to be better than that of pure ZnO and TiO_2_ (Degussa P25). When the nanoparticle- based catalyst was recycled and reused for subsequent disinfection experiments, its efficiency did not change remarkably, even after three cycles. The sonochemically synthesized Ag@ZnO core-shell nanoparticles thus have a good potential for application in solar photocatalytic disinfection of water borne pathogens.

## 1. Introduction

A large part of the population of developing countries is vulnerable to water borne diseases caused by pathogenic microbes present in the aquatic environment. Amongst the various enteric pathogens, *Escherichia coli* and *Staphylococcus aureus* are causal agents of various types of infections [1] and may lead to deterioration in the quality of drinking water in rural areas. The major causes behind this can be attributed to unawareness about personal hygiene practices and poor sanitation facilities. For disinfection of these microbes, composite nanoparticles-assisted photocatalysis has good potential for field application [2]. Currently, the simplicity and cost-effectiveness of sunlight- assisted photocatalysis using metal/metal oxide nanoparticles is gaining much attention for water treatment applications [3]. However, although the nanosized catalysts were successfully developed, certain issues such as short-shelf life of the nanoparticle systems due to catalyst poisoning, decreased active surface area of the nanoparticle systems by surface doping, and the possibility of leaching of reactive metal ions into the purified water have restricted their commercial exploitation. To deal with these limitations, core-shell structure nanoparticles were proposed. It is expected that nanoparticles with core-shell morphology will not only protect the metal catalysts, but also show promising results with regards to increased photocatalytic disinfection efficiency and extended shelf life of the material [4]. Metal@ZnO core-shell structure nanoparticles have been used for photocatalytic degradation of the organic dyes Rhodamine B and methyl orange in an aqueous solution [5,6]. *E. coli* has been extensively used as a good model micro-organism for studying photocatalytic disinfection but studies with *S. aureus* are mostly done with TiO_2_ and its doped variants [7,8]. To the best of our knowledge, no such disinfection with core-shell nanoparticles has been carried out with the latter micro-organism. Ag/TiO_2_ nanocomposites were previously explored for successful photocatalytic disinfection of *E. coli* [9]. Most of these reports have followed precipitation technique for coating metal oxide shell on noble metal (e.g., gold) nanoparticles. Although these materials have shown interesting photocatalytic properties, the high cost of gold is expected to hinder their practical application. Hence, Aguirre et al. tried to replace the gold core by a cheaper alternative, i.e., silver, and used this material for degradation of dyes [10]. Das and co-workers for the first time applied Ag@ZnO nanoparticles synthesized by a chemical precipitation technique for sunlight-assisted photocatalytic disinfection of the pathogenic bacterium *Vibreo cholerae* in synthetic as well as real water systems [11]. This could be a potential alternative to conventional disinfection techniques such as chlorination which are known to generate toxic byproducts [12]. However the conventional precipitation technique employed for the synthesis of metal@ZnO core-shell nanoparticles could not provide the well dispersed and porous materials required for catalytic applications [13]. Thus, it was necessary to investigate alternative synthetic protocols to obtain the monodispersed metal@ZnO core-shell nanoparticles and to check the potential of such materials for photocataytic applications. Recently, sonochemical techniques have been used extensively to obtain well dispersed and highly crystalline nanomaterials [4,14]. However, to the best of our knowledge, such techniques have never been exploited for the synthesis of metal@ZnO core-shell nanoparticles and examining their potential to disinfect bacterial pathogens. In the present paper, we report sunlight-assisted photocatalytic disinfection of two water borne pathogenic bacteria, *Escherichia coli* and *Staphylococcus aureus*, in saline solution (0.9%) and some real water systems using Ag@ZnO core-shell nanoparticles synthesized using an ultrasound assisted method.

## 2. Materials and Methods

### 2.1. Materials 

All the reagents and chemicals used in the synthesis of photocatalyst as well as in the disinfection reaction were of research grade (99.99% pure) and were procured from MERCK (Mumbai, India). De-ionized water was used during all synthesis processes.

### 2.2. Synthesis and Characterization of Ag@ZnO Core-Shell Nanoparticles 

Ag nanoparticles were synthesized by reduction of silver perchlorate monohydrate (Sigma-Aldrich, St. Louis, MO, USA) by NaBH_4_ and trisodium citrate dihydrate [11]. Experimental procedures were as follows: distilled water (97 mL) was placed in a 250 mL glass beaker which was placed in an ice bath. Silver perchlorate monohydrate (1 mL, 1 mM) followed by 100 mM sodium borohydride (1 mL) and 3 mM of trisodium citrate (0.885 mL) were added to the beaker under vigorous stirring. A transparent bright yellow color was observed immediately due to the formation of the Ag nanoparticles. This colloid was aged for 12 h at room temperature. Zinc oxide nanoparticles were coated on the surface of Ag nanoparticles via an ultrasound assisted precipitation technique. To zinc nitrate hexahydrate aqueous solution of a known concentration (50 mL), sodium hydroxide solution (1 M) was added to obtain a white precipitate of zinc hydroxide, which was redissolved by adding excess of sodium hydroxide. This solution (20 mL) was added to the aqueous dispersion of Ag nanoparticles (10 mL) and exposed to ultrasound for 30 to 90 min. Then the solution was allowed to cool by natural process. The composite nanoparticles were then collected by centrifugation (at 12000 rpm) and dried at 80 °C for 12 h. Following this, the nanoparticles were sintered at 200 °C and 400 °C for 1 h. During centrifugation nanoparticles were washed with de-ionized water (three times) to remove the water soluble sodium chloride and other impurities.

Formation of Ag nanoparticles, and Ag@ZnO core-shell nanoparticles was investigated by UV-visible spectroscopy (Carry 100, Agilent, Santa Clara, CA, USA) respectively. Morphology and crystal structure of the nanoparticles was analyzed by transmission electron microscopy (JEM-2010, JEOL, Akishima, Tokyo, Japan) and X-ray diffraction (XRD, Rigaku, Tokyo, Japan) techniques respectively. The composition/functional property of nanomaterials were analyzed with FTIR spectroscopy at room temperature in an acquired range of 500–4000 cm^−1^. Average surface area and porosity was measured by the Brunauer–Emmett–Teller (BET) technique. 

### 2.3. Preparation of Bacterial Cultures

Bacterial strains of the Gram negative bacterium *E. coli* DH5-alpha and Gram-positive bacterium *S. aureus* were used as the target microorganisms in this study. The strains were purchased from the Microbial Type Culture Collection and Gene Bank (MTCC, Chandigarh, India). The strains were grown aerobically in a nutrient broth (HiMedia, Mumbai, India) at 37 °C in a shaking incubator (Daihan Labtech, New Delhi, India) at 200 rotations per minute (rpm). At optical density (OD_600_) 0.6 for *E. coli* and 0.8 for *S. aureus*, corresponding to $10^{8}$ CFU/ml (CFU = colony forming unit), the bacteria were harvested by centrifugation at 5000 rpm for 10 min. They were thereafter washed with 0.9% normal saline solution (NSS) to provide appropriate osmotic conditions [11]. All the glassware and plastic-ware used for media preparation, experimental purposes and analysis were sterilized by autoclaving at 121 °C, for 20 min before being used [15].

### 2.4. Photocatalytic Disinfection Experiments 

Bacterial cells with a final cell concentration of 5 $\times 10^{6}$ CFU/mL were put in 1 L of normal saline solution and multiple reactions were performed with varying concentrations of Ag@ZnO ranging from 1 to 5 mg/L. Photocatalytic disinfection reactions were carried out in 2 L reactor vessels under continuous and controlled agitation (500 rpm).The set up was kept under dark conditions for 30 min to attain equilibrium. After the dark phase, the system was exposed to sunlight for 120 min and samples were collected at 15 min intervals. To monitor and analyze the inactivation of microbes, 100 μL of collected samples were further diluted in 900 µL of sterile 0.9% NSS and a volume of 100 μL from the final diluted sample was spread on nutrient agar plates. The plates were left for overnight incubation at 37 °C. Following this, viable cell count was performed to obtain the results for the rate of disinfection [11,16]. The above steps were repeated using two commonly used catalysts ZnO and TiO_2_ (Degussa P25) for comparative studies and with the optimum catalyst concentration for proper disinfection as obtained by Ag@ZnO. Additionally two experimental controls were performed. (1) In light control, under only photolytic condition the microbial population was exposed to sun-light in absence of Ag@ZnO. (2) In dark control, microbial population was reacted with Ag@ZnO in absence any light. The Intensity of sunlight was measured by a digital lux meter and found to be 90,000 ± 5000 lux. To evaluate whether the sun-light/Ag@ZnO assisted photocatalytic disinfection system is applicable to natural water systems, samples of tap (municipal supply, Bhubaneswar, India), river, and pond water were collected. Results were compared with de-ionized water. All water samples were collected and transported in clean and autoclaved sample bottles (Tarsons, Kolkata, India) at 4 °C and immediately were filtered by using Whatman filter paper and centrifuged at 5000 rpm for 15 min to remove insoluble materials followed by autoclaving to eliminate any microbial contamination. To demonstrate the efficiency of the synthesized catalyst, a calculated amount (as mentioned earlier) of targeted pathogens were spiked in the sterilized natural water samples and subjected to photocatalysis in presence of three different photocatalysts (Ag@ZnO, ZnO and TiO_2_). The concentration of catalyst used was the one which was obtained as the optimum for the respective bacteria from experiments conducted in saline solution.

### 2.5. Determination of Lipid Peroxidation

Malondialdehyde (MDA) is an end product of lipid peroxidation. Therefore estimation of MDA through its reaction with thiobarbituric acid (TBA), forming a pink colored MDA-TBA complex, predicts the disintegration or, rather the damage of microbial cell membrane leading to death [16,17,18]. To establish this, analysis was performed by obtaining 1 mL samples from the reactor contents at regular time intervals (5 min) and the samples were mixed with 2 mL of 10% (wt./vol.) trichloroacetic acid. The mixture was subjected to centrifugation at 11,000 *g* for initial 35 min and then again for an additional 20 min to ensure the removal of precipitated proteins, catalyst, cells and other possible solid components from the system [16]. 3 mL of freshly prepared 0.67% (wt./vol.) TBA (Sigma Aldrich) was added to the supernatant. The samples were boiled in a water bath for 10 min and then the absorbance was measured at 532 nm using UV-visible spectrophotometer. The concentration of MDA in the system was calculated in nanomoles of MDA released per mg dry weight of bacteria [18].

### 2.6. Potassium Ion (K^+^) Leakage Studies

To study the K^+^ leakage from photocatalytically inactivated bacteria, 2 mL sample was collected at regular time intervals (2 min) from the reaction system and was subjected to centrifugation as per the details given in previous reports [18,19]. The supernatant was collected and analyzed using microwave plasma atomic emission spectrometer (4200 MP-AES, Agilent Technologies, Santa Clara, CA, USA).

### 2.7. Stability and Reusability of the Photocatalyst 

The stability of the catalyst in post reaction condition was investigated using XRD. Additionally for further confirmation the post reaction water sample was analyzed using MP-AES to detect the leaching of Ag^+^ and Zn^2+^ ions during the photocatalytic disinfection experiment [11]. Catalyst was recovered by centrifugation at 12,000 rpm for 30 min and dried at 80 °C and reused for the photocatalytic disinfection application. Unless otherwise mentioned all the experiments were conducted in triplicate.

## 3. Results and Discussion

### 3.1. Characterization of Nano-Photocatalyst

**Figure 1:**
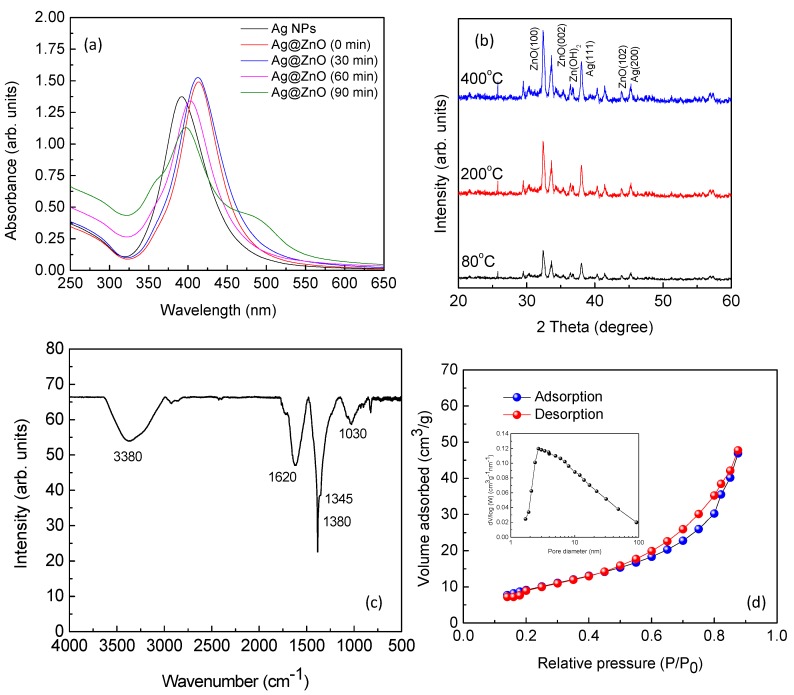
UV-Visible spectra of aqueous dispersion of Ag and Ag@ZnO core-shell nanoparticles (**a**), XRD pattern (**b**) and FTIR spectrum (**c**) of Ag@ZnO core-shell nanoparticles, (**d**) Nitrogen adsorption/desorption isotherms obtained at 77 K and inset shows the pore size distribution of the as-synthesized Ag@ZnO NCs synthesized by the sonochemical technique and dried at 80 °C for 2 h.

UV-visible spectra of the aqueous dispersion of Ag nanoparticles, and Ag@ZnO core-shell nanoparticles synthesized by ultrasonic hydrolysis of zinc nitrate hexahydrate are shown in Figure 1a. Aqueous dispersion of Ag nanoparticles showed a clear SPR band at 391 nm which showed a distinct red shift of about 22 nm immediately after addition of aqueous sodium zincate sol. This is attributed to an immediate change in the chemical environment around Ag nanoparticles. With increase in the ultrasonic irradiation time SPR band has shown a red shift to 397 nm with development of a shoulder peak at 487 nm. It is expected that during the formation of core-shell nanoparticles, Ag nanoparticles may have aggregated slightly to form large clusters. This may have caused dipole coupling between closely interacting metal nanoparticles. This hypothesis is supported by the electron microscopy images.

Results of our XRD study is shown in Figure 1b. For the synthesized nanoparticles, three distinct peaks at 2θ = 38.2, 44.9 and 64.8 corresponding to (111), (200) and (220) planes of metallic Ag with face-centered cubic structure (JCPDS Card No. 04-0783) is observed. Similarly three major peaks of ZnO at 2θ = 31.99, 34.63, and 46.51 corresponding to (100), (002), and (102) planes of synthetic ZnO with hexagonal wurtzite structure (JCPDS Card No. 36-1451) are obtained. Any peak corresponding to other Ag/Zn compounds was not obtained. This suggests that no alloy or solid solution is formed. Mean crystallite diameter (MCD) was found to be ≈ 15 and 25 nm for Ag and ZnO nanoparticles respectively. It is also observed that the crystal structure and phase remained unchanged after heat treatment (at 200 and 400 °C). However the MCD and crystalinity have increased slightly after heat treatment. The results of FTIR spectroscopy are shown in Figure 1c. The broad band around 3400 cm^−1^ may correspond to O–H stretching mode of hydroxyl groups whereas the strong peak at 2345 cm^−1^ resembles to the stretching mode of acidic O–H group, which arises in the range of 2400–3300 cm^−1^. The small vibration appearing at 1630 cm^−1^ may belong to the stretching peak of C=O group [11]. Vibration peaks at 1500 and 1280 cm^−1^ corresponds to C–H bending and C–O stretching mode respectively [11,14]. The peaks at 1630 and 637 cm^−1^ may correspond to Zn–O stretching and deformation vibration, respectively [14].

The adsorption-desorption isotherm plot for the nitrogen sorption (77 K) of the Ag@ZnO nanoparticles sample that was synthesized by sonochemical technique and dried at 80 °C for 2 h shows typical “type IV” isotherm in the Brunauer classification (Figure 1d). The sample exhibited average pore size in the range of 5–20 nm indicating the porous nature of the material. The specific surface area of Ag@ZnO core-shell nanoparticles was evaluated to be 65.5 m^2^/g based on the BET result. This high surface area and porous nature are expected to be very beneficial for photocatalytic applications [12,14].

**Figure 2:**
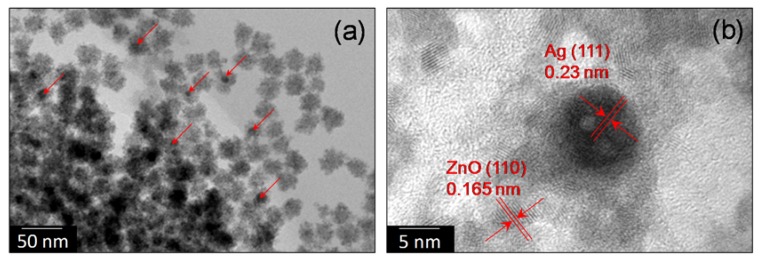
TEM (**a**) and HRTEM (**b**) images of Ag@ZnO core-shell nanoparticles synthesized by the sonochemical technique and dried at 80 °C for 2 h.

Morphology of the Ag@ZnO synthesized by the sonochemical technique were investigated by TEM. TEM samples were prepared by dipping the TEM grid in aqueous colloidal dispersion of NC followed by freeze drying for 12 h. Figure 2 shows TEM and HRTEM images of core-shell Ag@ZnO nanoparticles. Core-shell structure is observed for the materials. However, multiple silver nanoparticles were encapsulated within a single zinc oxide shell. Similar situation was also observed by Tripathy et al. [11]. A broad size distribution is observed for synthesized nano-Ag particles. The size of the Ag is found to be in the range of 10–30 nm and that of ZnO shell is about 5 to 10 nm. Metal core was found to have inter planar spacing of ~0.23 nm which corresponds to the (111) plane of the metallic silver with face-centered cubic structure. In ZnO shell, the spacing between adjacent lattice fringes is 0.16 nm, which is close to the *d-*spacing of the (110) plane of hexagonal ZnO (exact value is 0.168 nm). 

### 3.2. Photocatalytic Disinfection of Target Pathogens

**Figure 3:**
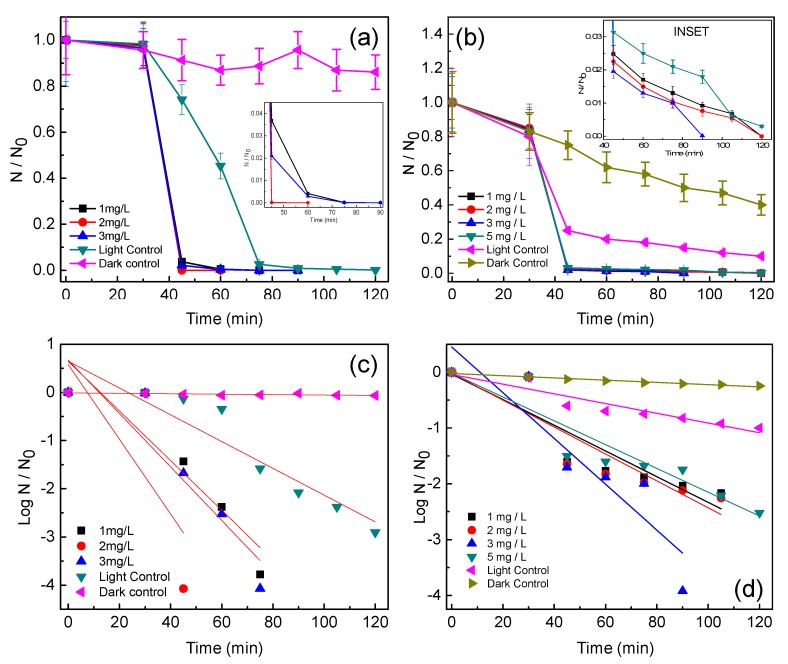
Effect of Ag@ZnO core-shell NPs loading on the solar-PCD kinetics of (**a**) *E. coli* and (**b**) *S. aureus*. Linear fitting plots of PCD kinetics of (**c**) *E. coli* and (**d**) *S. aureus* according to Chick-Watson model. Initial bacteria concentration = 5 × 10^6^ CFU/mL, Temperature = 35 ± 2 °C. Error bars indicate the standard deviation of replicates (*n* = 3).

**Figure 4:**
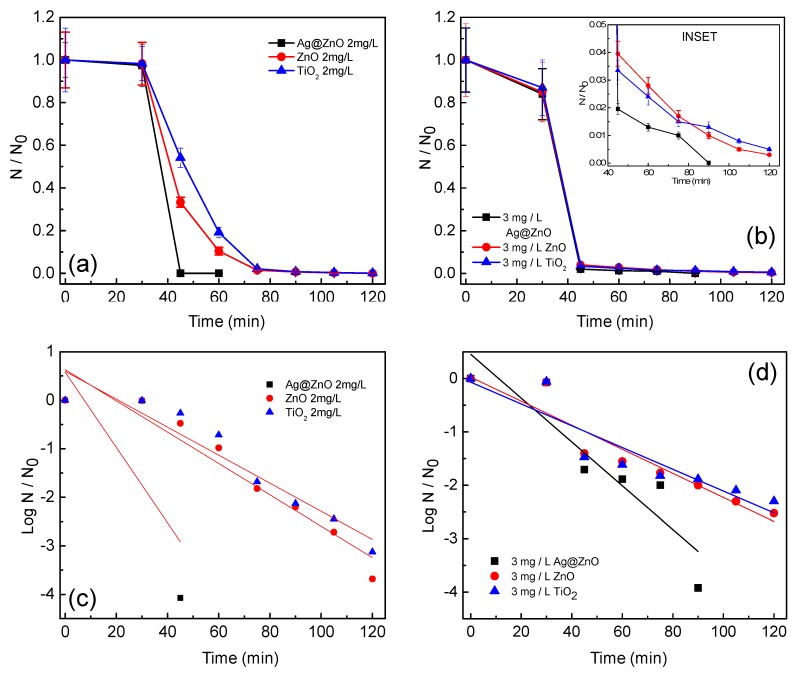
Effect of different catalysts on the solar-PCD kinetics of (**a**) *E. coli* and (**b**) *S. aureus* at a catalyst loading of 2 mg/L and 3 mg/L respectively. Linear fitting plots of PCD kinetics of different catalysts against (**c**) *E. coli* and (**d**) *S. aureus* according to Chick-Watson model at a catalyst loading. Initial bacteria concentration for each experiments = 5 × 10^6^ CFU/mL, Temperature = 35 ± 2 °C. Error bars indicate the standard deviation of replicates (*n* = 3).

Figure 3a,b show the photocatalytic disinfection achieved against the target pathogens at different catalyst concentrations. In Figure 3c,d bacterial disinfection is represented in its corresponding log reduction profile and the disinfection pattern is validated through comparison of the obtained profile with the standardized Chick-Watson model [11,20,21]. Figure 3a,b suggest that amongst the concentrations tested, 2 mg/L and 3 mg/L resulted in complete disinfection (6 log reductions) of *E. coli* and *S. aureus*, respectively, in 60 min and 90 min, respectively. It is observed that sunlight alone is not effective for the complete disinfection of the targeted pathogens as only 3 and 2.5 log reductions could be observed for *E. coli* and *S. aureus*, respectively, at 120 min. Experiments conducted under dark conditions did not show any remarkable change in the microbial colony counts, as less than 0.5-log reduction for both the microorganisms was achieved in 120 min (Figure 2c,d). Using the optimum concentration of Ag@ZnO nanoparticles for each of the bacteria for photocatalytic disinfection, comparative sunlight-assisted photocatalytic disinfection activity was evaluated with pure-ZnO and commercial TiO_2_ (Degussa P25) and the results are shown in Figure 4a,b. Figure 4c,d shows the Chick-Watson disinfection kinetics of *E. coli* and *S. aureus* using different photocatalysts. These results suggest the superior disinfection efficiency of Ag@ZnO nano-photocatalyst compared to the conventional metal oxide systems. An increase in inactivation for both targeted bacteria was observed with the increase in catalyst concentration from 1 to 2 mg/L in *E. coli* and 1 to 3 mg/L in *S. aureus*. With further increase in the catalyst concentration beyond the mentioned range, a deterioration in disinfection rate was obtained for both the targeted microorganisms. With lower concentration of catalyst the amount of reactive oxygen species (ROS) generated is comparatively less. Thus complete disinfection required a longer irradiation time [22]. It is expected that as the rate of ROS production is slow at lower concentrations of catalyst, and under the initial conditions the microorganisms may activate their molecular resistance mechanisms. Therefore an extended disinfection time period is required for sufficient ROS generation and thus under the constant attack of ROS, bacteria may lose their capability of reactivation. With an increase in catalyst concentration the ROS generation rate increases, which is expected to improve the disinfection rate. Similarly, under the optimal conditions, the rate of ROS generation is maximum and therefore it may be expected that the interaction of the same with bacterial cells is more frequent. This may lead to an enhanced disinfection rate. It is further noticed that with increase in the catalyst concentration, disinfection gets delayed. This is mainly because with the increase in catalyst concentration the turbidity of the system increases, thereby blocking the sunlight irradiation from uniformly reaching the catalyst particles and cells, hence resulting in slower inactivation [23]. The current study involves *E. coli* and *S. aureus* bacteria. The photocatalytic performance of a photocatalyst depends both on its concentration and the irradiation time. *E. coli* was found more sensitive to sunlight-assisted photocatalytic disinfection process than *S. aureus*, as it requires comparatively less catalyst concentration and shorter sunlight irradiation time in comparison to *S. aureus* as evidenced from Figure 3a,b. The difference in susceptibility of both bacterial species to Ag@ZnO nanoparticles can be ascribed to the differences in their cell membrane/wall structures, chemical components, biological shape, and differences in robustness of Gram-positive and Gram-negative bacteria [24].

**Figure 5:**
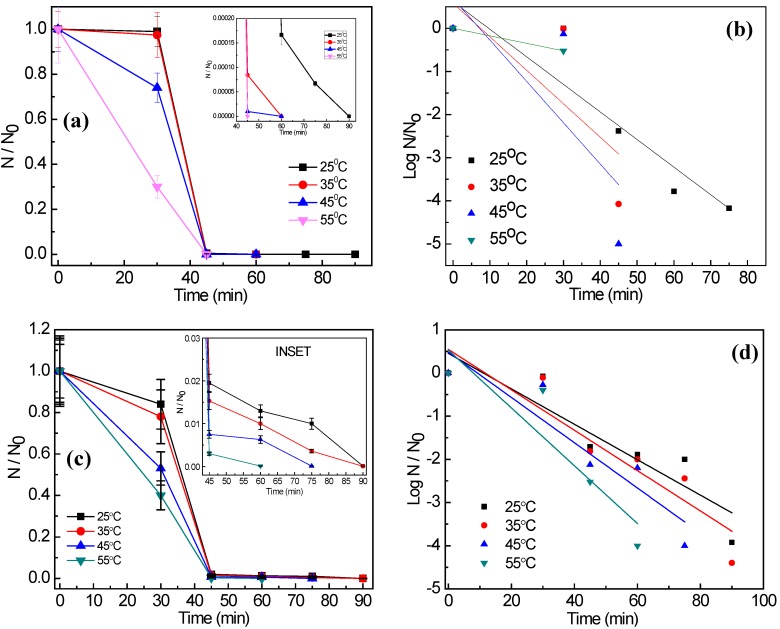
Effect of different reaction temperature on the solar-PCD kinetics of (**a**) *E. coli* and (**c**) *S. aureus* at a catalyst loading of 2 mg/L and 3 mg/L respectively. Linear fitting plots of PCD kinetics of different reaction temperature against (**b**) *E. coli* and (**d**) *S. aureus* according to Chick-Watson model. Initial bacteria concentration for each experiments = 5 × 10^6^ CFU/mL, Error bars indicate the standard deviation of replicates (*n* = 3).

From Figure 4, it is observed that Ag@ZnO nanoparticles show enhanced disinfection efficiency for both targeted pathogens in comparison to the classical metal oxide systems (ZnO and TiO_2_). The expected reason behind the enhanced efficiency may be the positioning of the noble metal (i.e., Ag) in the core and encapsulating it with a ZnO shell. Photocatalytic disinfection involves the excitation of the photocatalyst with light energy greater than or equal to that of the band gap [25]. On excitation the electrons forming the valence band of the metal oxide shuttle to the conduction band, where they are usually accepted by electron acceptors present in the reaction environment. This reduction pathway leads to the formation of ROS which results in killing of microbial cells by damaging their membrane integrity [24,26]. Therefore it leads to subsequent release of the intra-cellular components, which become vulnerable to the ROS attack [26,27]. Figure 5a–d show the effect of temperature on the photocatalytic disinfection of the targeted pathogens. These results show that as the temperature increased, a maximum process efficiency was observed at a reaction temperature of 55 °C. It is thus observed that, the rate of disinfection improved as the temperature of the reaction system increased. At 55 °C disinfection is achieved within 45 min and 60 min for *E. coli* and *S. aureus*, respectively. The post-disinfection reactivation of the target microbes was monitored for 24 h. None of the microbes showed an6y reactivation thus suggesting cell death due to damage caused by the ROS to both the target pathogens.

### 3.3. Determination of MDA to Study the Membrane Lipid Peroxidation

**Figure 6:**
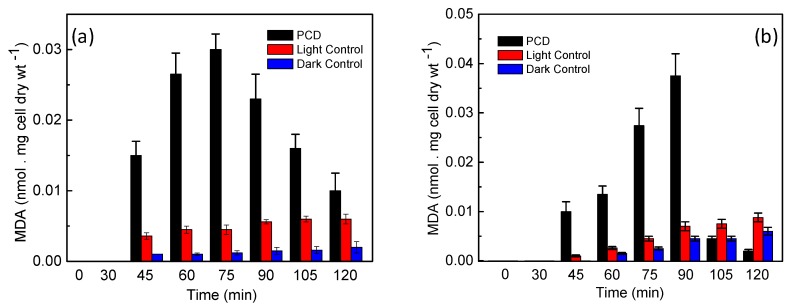
Lipid peroxidation kinetics of (**a**) *E. coli* and (**b**) *S. aureus* cells subjected to solar-photocatalysis in presence of 2 mg/L and 3 mg/L Ag@ZnO NPs respectively. Initial bacteria concentration = 5 × 10^6^ CFU/mL, Temperature = 35 ± 2 °C. Error bars indicate the standard deviation of replicates (*n* = 3).

Time dependent generation of MDA (a key biomarker of membrane lipid peroxidation) for *E. coli* and *S. aureus* subjected to photocatalytic disinfection under their respective optimum catalyst concentration and temperature of 35 °C is shown in Figure 6a,b. Earlier experiments had shown complete disinfection at 60 and 90 min, respectively, for *E. coli* and *S. aureus* (Figure 3a,b). Hence, a similar correlative result can be inferred from the above mentioned figure. It is quite evident that maximum generation of MDA is observed after 75 min i.e., 0.03 nmol/mg cell dry weight and 90 min i.e., 0.0375 nmol/mg cell dry weight for *E. coli* and *S. aureus* respectively, which indicates cell membrane disintegration resulting in disinfection. Slight elevation in MDA production is seen within the first 30 min, which may be attributed to a loss of membrane integrity due to the action of shear stress produced on the microbial cells due to the continuous stirring conditions [28]. Additionally, the misbalance of ionic potential may also play a role in loss of membrane integrity leading to lipid peroxidation. It may also be noted that after the reported disinfection time, a decline in MDA concentration has been initiated. After a threshold level of MDA is generated in the photocatalytic system, it is expected to be mineralized being an organic compound itself [18,29]. When the microbial cells were exposed to sunlight without the presence of photocatalysts, less than even 0.01 nmol/mg cell dry weight generation was observed in both the microbes as shown in Figure 6a,b. However, the effect of Ag@ZnO on microbial cells without the presence of light is also found to be non-substantial, where the concentration was less, as 0.005 nmol/mg cell dry weight were quantified for both the test microbes. It is proposed that generation of ROS (such as OH^•^ radical) in the photocatalytic process may lead to peroxidation of the cell membrane peptidoglycan layer and membrane proteins, followed by decomposition of cellular components and cellular disintegration [16,17,18,19,29], as ROS mainly (•OH) hit unsaturated membrane lipids to make lipid radicals. This, in the presence of oxygen is expected to give a lipid peroxyl radical capable of abstracting hydrogen from an adjacent unsaturated lipid and produce a lipid hydroperoxide and a lipid radical. This series of reactions continues until all the membrane unsaturated lipids are destroyed and malondialdehyde (a stable by-product of membrane lipid peroxidation) is subsequently produced. MDA generation patterns suggest that lipid peroxidation in *E. coli* maintains a uniform rate while a sporadic rate occurs for *S. aureus*, thus suggesting a higher robustness of the latter in comparison to the former [24].

### 3.4. Analysis of Potassium Ion (K^+^) to Study the Cell Membrane Damage

**Figure 7:**
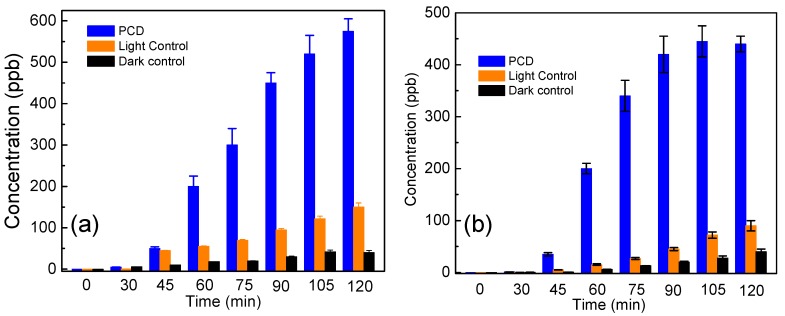
Leakage of K^+^ ion from (**a**) *E. coli* and (**b**) *S. aureus* cells subjected to solar-photocatalysis in presence of 2 mg/L and 3 mg/L Ag@ZnO core-shell NPs, respectively. Initial bacteria concentration = 5 × 10^6^ CFU/mL, Temperature = 35 ± 2 °C. Error bars indicate the standard deviation of replicates (*n* = 3).

Leakage of K^+^ ions is generally considered as a dominating evidence of compromised cellular integrity. The results obtained through K^+^ leakage analysis are in agreement with many previous studies which mention the dysfunction of potassium channels of microorganisms on photocatalytic treatment [18,30,31,32]. It can be observed that the concentration of K^+^ (in ppm) increases with the increase of the reaction time up till a particular time period beyond which the concentration in the reaction environment becomes constant. As shown in Figure 7a,b the maximum K^+^ estimated after 120 min for *E. coli* and *S. aureus* after photocatalytic disinfection was found to be 575 ppb and 440 ppb, respectively. An interesting observation was made that the time required for complete disinfection for each of the target bacterium, as evaluated from the decreasing CFU count, does not correspond well with the K^+^ leakage pattern. This must be because the primary target of photocatalytically produced ROS is membrane lipids. Once the entire membrane of the bacteria is compromised, an increase in K^+^ ion is expected. This pattern of K^+^ release suggests that the increase in the concentration of potassium ion in the reaction environment indicates a steady progress in the photocatalytic disinfection process. Once the entire bacterial death is achieved, it is expected that the total amount of K^+^ will be maintained for the remaining reaction phase [33]. 

### 3.5. Stability and Reusability of the Catalyst Post Disinfection

**Figure 8:**
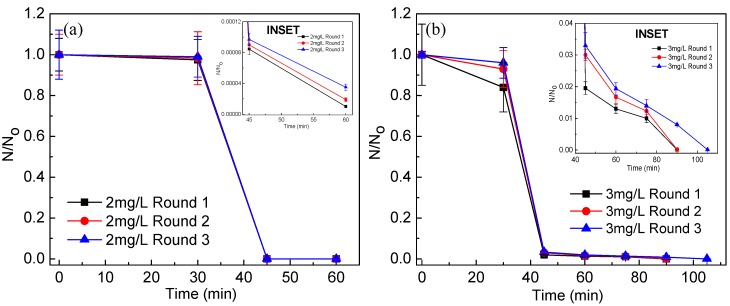
Effect of Ag@ZnO core-shell NPs reusability till three rounds of solar-PCD kinetics of (**a**) *E. coli* and (**b**) *S. aureus*. Initial bacteria concentration = 5 × 10^6^ CFU/mL, Temperature = 35 ± 2 °C. Error bars indicate the standard deviation of replicates (*n* = 3).

When the stability of the catalyst in post-reaction condition was investigated using XRD, no alteration in the crystal structure of Ag@ZnO was observed, suggesting its structural stability throughout the process [11,34]. It is known that leaching of material could re-toxify the system and it could also be argued that Ag^+^ and Zn^2+^ ions which are reported to show antimicrobial properties may leach out of the system and hence, may be the actual cause of disinfection. However, the answer to this possibility is already communicated in our previous paper [11], there being no detectable amount of Ag^+^ and Zn^2+^ ions in the system post-disinfection. If the catalyst could be recycled after photocatalytic disinfection then it may be suitable for commercial exploitation of the process. Ag@ZnO core-shell nanoparticles were recycled after the photocatalytic disinfection experiments and used for next batch of bacterial disinfection experiment (after heating at 80 °C). As shown in Figure 8, core-shell nanophotocatalyst exhibited insignificant reduction in *E. coli* and *S. aureus* disinfection efficiency, even after three consecutive cycles.

### 3.6. Photocatalytic Disinfection Efficiency in Real Water Systems

**Figure 9:**
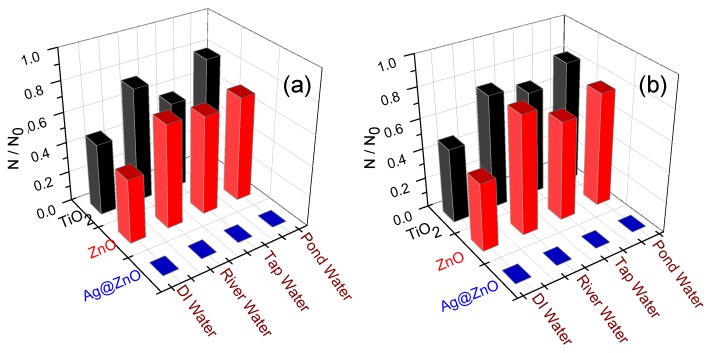
Effect of different photocatalysts on the relative reduction in the (**a**) *E. coli* and (**b**) *S. aureus* cell count (*N/N_0_*) in real water samples after 120 min of solar irradiation at a catalyst loading of 2 mg/L and 3 mg/L catalyst concentration, respectively. In each case the initial bacteria concentration = 5 × 10^6^ CFU/mL, Temperature = 35 ± 2 °C.

As the results show (Figure 9), Ag@ZnO exhibits a better disinfection profile as compared to pure semiconductors in case of all the real water samples. The results correspond well with our previous results [11]. The superiority of the Ag@ZnO as compared to the traditional photocatalysts can be attributed to many causes. It is a well-known and established fact that the photocatalyst that is being used here has a core shell nanocomposite structure. The structure itself has many advantages over its traditional counterparts. It is a matter of general observation that the metal ions in the composite structure are protected by the shell in the composite structure. This has many advantages: firstly it solves the problem of leaching out of the silver metal ion. Silver is itself a very poisonous metal ion and detrimental and harmful to various organisms [4,5,11]. At the same time, the target pathogens *E. coli* and *S. aureus* are unable to survive and escape its effects. The core shell morphology also increases the surface area of the photocatalyst. As the surface area increases, so does the effectivity of the photocatalyst. Both the traditional photocatalysts used here, namely TiO_2_ and ZnO lack in this property. The lack of a proper nanocomposite structure in the cases of TiO_2_ and ZnO can also explain the lesser efficiency that these photocatalysts show in the photocatalytic degradation of real water samples.

Various studies have already shown that at various concentrations both zinc and silver are detrimental to the growth of microorganisms [35]. The photocatalyst that we have used contains both these elements, so as a result, a better result can always be expected than that from the traditional ones, namely the likes of ZnO and TiO_2_. The combined effect of toxicity of these two potent antimicrobial agents, combined with the lesser amount of leaching due to the unique structure is indeed a deciding factor in increasing the efficiency of the photocatalyst against the traditional players [11].

However, the issue of safety can be raised, regarding the compatibility of silver and zinc in various water streams and water bodies, as both of these metals are known to be toxic to organisms [36,37]. To attend these sensitive issues, we did an MP-AES assay, and it was observed that the concentration of zinc and silver was below the detectable levels. Thus it addresses most of the toxicity-related issues.

### 3.7. Proposed Mechanism of Photocatalytic Disinfection

**Figure 10:**
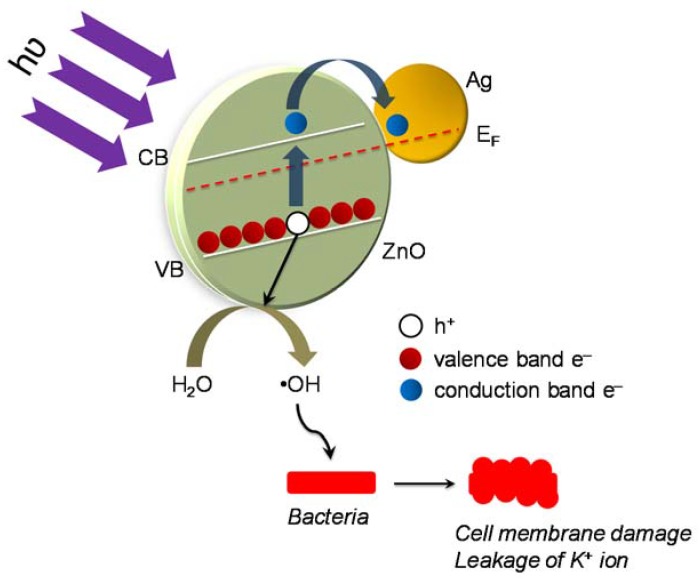
Proposed mechanism of sun-light assisted photocatalytic disinfection of bacteria using Ag@ZnO core-shell nanoparticles

The possible disinfection mechanism has been reported in the literature [9,11,15]. In the present case, we expected that the disinfection mechanism is contributed by the action of the photo-induced reactive oxygen species generated during the reaction (Figure 10). The initial site of attack is expected to be the lipopolysaccharide layer present in the external cell walls of the target bacteria [11]. It is assumed that the oxidative stress which is generated due to this process disintegrates the peptidoglycan layer and results in peroxidation of the lipid membrane, eventually causing oxidation of the membrane proteins [9]. This leads to rapid leakage of K^+^ ions from the bacterial cells hence dysfunction of the potassium channels resulting in deregulation of cell signaling. Additionally the dwindling cell functionality and viability is also attributed by the peroxidation of polyunsaturated phospholipid components of the cell membrane, eventually leading to cell death [11,15].

There is an increasing demand regarding the issue of providing safe and potable drinking water to underdeveloped Third World countries. There is an urgent need to develop strategies that follow an alternate route to address this concern [18]. Based on the above statement, the concept of “Advanced Oxidation Process” can be proposed; based on the proven effectivity and superiority as compared to that of other traditional catalysts.

The catalyst that we have proposed, generally works well towards the basic range of pH values. All the real water samples, especially the likes of tap-water, and river water have basic pH. This can also be explain the better effectivity and working efficiency of the proposed catalyst, although further confirmation is required.

It can also be concluded from the MP-AES analysis that the proposed catalyst is completely non-toxic in nature and can be applied for a wide range of applications. It can also be concluded that, since there is no evidence for the proposed catalyst’s toxicity to organisms, it can surely be used as a better, safer option than the traditional ones. 

## 4. Conclusions 

When DI water contaminated with *E. coli* and *S. aureus* was subjected to Ag@ZnO core-shell nanoparticles mediated photocatalytic disinfection under sun-light radiation, complete disinfection of *E. coli* and *S. aureus* was achieved within 60 and 90 min respectively at 35 °C and in 45 and 60 min at 55 °C. Quantitative analyses of K^+^ ion release and MDA assay proposed the damage of bacterial cell wall by ROS generated during solar photocatalysis. The disinfection profile for both the bacteria was validated using the Crick-Watson disinfection model. Disinfection achieved using the Ag@ZnO system was also validated for real world samples of municipal tap, pond and river water. When the nanocatalyst was recycled and reused for subsequent photocatalytic disinfection experiments, its efficiency did not change remarkably, even after three cycles. The reportted photocatalytic system may find applications in designing a portable water decontamination system for pathogen infested geographical locations.
